# Role of allogeneic hematopoietic cell transplantation in VEXAS syndrome

**DOI:** 10.1007/s00277-024-05942-2

**Published:** 2024-08-22

**Authors:** Ajoy L. Dias, Emma M. Groarke, Dennis Hickstein, Bhavisha Patel

**Affiliations:** 1 Immune Deficiency - Cellular Therapy Program, National Cancer Institute, National Institute of Health, Building 10 CRC/Room 3-3150, 10 Center Drive MSC 1102, Bethesda, MD 20892 USA; 2 Hematology Branch, National Heart, Lung, Blood Institute, National Institute of Health, Bethesda, MD USA

**Keywords:** VEXAS syndrome, Myelodysplastic syndrome (MDS), Allogeneic stem cell transplantation (Allo-HCT), Janus Kinase (JAK) inhibitors

## Abstract

VEXAS (vacuoles, E1 enzyme, X-linked, autoinflammatory, somatic) is a newly diagnosed syndrome comprising severe systemic inflammatory and hematological manifestations including myelodysplastic syndrome and plasma cell dyscrasia. Since its discovery four years ago, several groups have identified pleomorphic clinical phenotypes, but few effective medical therapies exist which include Janus Kinase (JAK) inhibitors, interleukin inhibitors (IL-1 and IL-6), and hypomethylating agents. Prospective trials are lacking at this time and most patients remain corticosteroid dependent. VEXAS has a high morbidity from frequent life threatening inflammatory symptoms and risk of progression to hematological malignancies and has an overall survival of 50% at 10 years. Allogeneic stem cell transplant (allo-HCT) is a curative option for this disease caused by somatic mutations in the *UBA1* gene. Here we outline the role of allo-HCT in treating patients with VEXAS syndrome, highlighting the outcomes from several single-institution studies and case reports. Prospective trials will be required to precisely define the role of allo-HCT in the management of VEXAS syndrome.

## Introduction

VEXAS syndrome is an X-linked, systemic, hemato-inflammatory somatic syndrome that was first reported in 2020 [1]. The acronym stands for *V*acuoles, *E*1 enzyme, *X*-linked, *A*utoinflammatory, and *S*omatic. It was first described in a cohort of 25 men who presented with a systemic autoinflammatory disease along with vacuoles in the hematopoietic precursors (a hallmark of the disease) and were found to harbor acquired mutations in the *UBA1* gene. *UBA1* gene encodes for the E1 enzyme involved in the first step of cellular ubiquitylation [2]. VEXAS occurs in older males and the syndrome consists of a constellation of autoinflammatory features often accompanied by hematological disease primarily macrocytic anemia and myelodysplastic syndrome (MDS). Although initially *UBA1* was thought to be 100% penetrant, recently in a large cohort, *UBA1* was shown to have incomplete penetrance and somatic mosaicism has been identified on next-generation sequencing (NGS) [3, 4].

There are no standard treatment protocols for this novel disease. Current treatment strategies include systemic steroids, Janus kinase [JAK] inhibitors, interleukin inhibitors, hypomethylating agents (HMA), and allogeneic stem cell transplant (allo-HCT). Because the medical therapies target the downstream inflammation and are not disease modifying, the only curative option appears to be an allo-HCT in eligible patients. However, allo-HCT is associated with an inherent risk of morbidity and mortality; thus, the benefits and risks of allo-HCT should be carefully weighted until therapeutic algorithms are established.

This brief review highlights the indications, rationale, and the role of allo-HCT in treating VEXAS syndrome. The current experience is limited, and the transplant regimens utilized differ among institutions. Thus, it will be essential to collect data in which patients received an upfront allo-HCT to be able to design future clinical trials.

## Pathophysiology

VEXAS syndrome is characterized by an acquired inactivating mutations in the X-linked *UBA1* gene [5]. These mutations are seen in the hematopoietic progenitor cells in the bone marrow and are lineage restricted to myeloid cells in circulation. *UBA1* codes for the main E1 activating enzyme in humans responsible for more than 90% of the activation of ubiquitin, ubiquitylation dependent intracellular protein degradation, and cell homeostasis [6, 7]. As *UBA1* is located on the X chromosome, VEXAS affects predominantly men, but rare cases of women with acquired or constitutional (Turner syndrome) X monosomy have been reported [8, 9]. Most *UBA1* mutations described so far affect the translation of *UBA1b*, the cytoplasmic isoform, either directly affecting the methionine 41 codon or generating an alternative splicing that skips methionine 41 [10, 11]. In the absence of methionine 41, an alternative initiation codon Met67 is used to start translation resulting in a novel smaller isoform of *UBA1c* which has a reduced catalytic activity to form ubiquitin thioester bonds [1]. Canonical mutations involve substitutions of methionine-41 (p.Met41); p.Met41Thr is the most common variant followed by p.Met41Val and p.Met41Leu [10–13]. The other less common mutations include splice acceptor site immediately preceding exon 3 affecting 6% leading to initiation at p.Met67 and expression of *UBA1c* [11, 13], Ser56Phe found in *UBA1* exon 3 resulting in reduced catalytic activity of *UBA1* [13, 14], and the recently identified six novel mutations in *UBA1* ( p.His55Tyr, p.Gly477Ala, p.Ala478Ser, p.Asp506Gly, p.Asp506Asn and p.Ser621Cys) which all are known to lead to VEXAS syndrome since none of them lead to *UBA1c* production [15].

Although the mutation is seen in multipotent hematopoietic progenitors, the expression of the mutated allele is seen in specific cell lines, mostly in the myeloid progenitors. Due to the mutations in *UBA1* in the monocytes, the free ubiquitin accumulates in the cells, and the accumulation of this misfolded protein is no longer cleared by the proteasome which then results in the activation of the unfolded protein response and autophagy [1]. The development of lymphopenia is part of the disease, however, the mutant allele is not present in the lymphocytes, implying a negative selection of mutated cells in lymphogenesis [16].

## VEXAS and bone marrow Failure/MDS

Ineffective hematopoiesis is evident in VEXAS patients initially as macrocytosis and progressive bone marrow failure presents as macrocytic anemia and thrombocytopenia. Bone marrow examination reveals characteristic cytoplasmic vacuoles in almost all patients as well as hypercellularity with myeloid hyperplasia and erythroid hypoplasia, however it needs to be noted the sole presence of vacuoles in myeloid precursors is not specific to VEXAS syndrome [17, 18]. Vacuolization of hematopoietic precursors is seen in other clinical conditions such as copper deficiency, zinc toxicity, alcohol abuse, antibiotic treatment and MDS. A threshold of 10% of myeloid precursors with > 1 vacuole had a high degree of sensivity and specificity for VEXAS [17–20]. Megakaryocytes are often dysplastic without meeting the criteria for MDS. The diagnosis of MDS is likely overestimated in earlier reports (50–60%) [1, 10, 13, 21], given the most recent criteria based on dysplasia. In a recent study, applying more stringent diagnostic criteria for MDS, the incidence was found to be closer to 20–30% [22]. Another study published recently by the group from Memorial Sloan Kettering Cancer Center (MSKCC)identified UBA1 mutations in 1% of MDS patients and inflammatory clinical presentation and vacuoles were observed in 83% and 71% respectively of patients with pathogenic *UBA1* mutations [23]. VEXAS MDS differs from classical MDS in terms of both heterogeneity and molecular landscape and occurs late in disease course (median, 4 years); it is uni- or multilineage dysplasia and rarely displays a severe prognostic score [21]. Most of the patients are in the low or intermediate risk according to the Revised International Prognostic Scoring System (R-IPSS, < 3.5) [24, 25]. The majority of patients have normal karyotype on conventional cytogenetics, and the mutational profile appears less complex. The few co-mutations described include DNMT3A, MLL, CSF1R, SF3B1, TET2, GNA11 and ZRSR2 and the significance of these mutations is unclear. In a recent analysis of 80 VEXAS patients, DNMT3A and TET2 mutations dominated the clonal landscape at discrepant variant allele frequency (VAF) but were not associated with hematologic or inflammatory manifestations [22]. The MSKCC study identified a median one additional myeloid gene mutation often in TET2, DNMT3A, ASXL1 or SF3B1 [23].

The pathogenesis of MDS remains elusive and investigational. There is a growing understanding that the highly inflamed bone marrow microenvironment may be playing a key role in its development. Whether clonal hematopoietic cells dysregulate the microenvironment to enhance their survival and suppress the normal hematopoiesis, or an altered microenvironment initiates MDS is not understood. The *UBA1* gene mutation could be the driving force for both these processes. In addition, there is a significant decrease in the peripheral dendritic cells and in monocyte subsets in patients with MDS, which could be due to increased apoptosis, or due to an impaired bone marrow production, or from redistribution of these cells to sites of inflammation [26].

The MDS in VEXAS syndrome is low/intermediate risk by the R-IPSS classification. The French VEXAS group has found the VEXAS MDS patients to have a higher symptom burden, lower platelet counts, and more frequent corticosteroid requirement than VEXAS alone patients [27]. They identified three phenotypic clusters; cluster one characterized by mild to moderate disease, cluster two which comprised MDS and higher mortality rates, and a third cluster representing an older age group, with constitutional symptoms, high C-reactive protein levels and less frequent chondritis. The 5-year probability of survival was 84·2% in cluster one, 50·5% in cluster two and 89·6% in cluster three. The *UBA1* p.Metgut41Leu mutation was associated with a better prognosis ( 100% [95% CI100;100]), compared to 76.7% (58.8;100.00) for p.M41Val (*p* = 0.04) and not different from p.Met41Thr with 83.1% (70.5;98.0)(*p* = 0.1). In another study of 80 VEXAS patients, presence of cytopenia(s), a diagnosis of MDS and clonal hematopoiesis particularly DNMT3A and TET2 at higher VAF predicted for an increased mortality risk [22].

Transfusion dependency occurs in 32% of all VEXAS MDS patients. This increases the risk of mortality by approximately 4.5-fold [25]. Even though VEXAS patients fulfil the diagnostic criteria of MDS, the risk stratification, clinical course and mortality does not correlate clearly with its seriousness; thus, there is the possibility that this could represent a separate hematological phenotype needing different prognostic criteria.

There are additional hematological manifestations in VEXAS. These include plasma cell dyscrasias reported in approximately 25% of patients, with several cases highlighting MGUS/MDS co-occurrence [1, 21] and rarely CMML [28]. VEXAS patients have an increased rate of unprovoked venous and arterial thromboembolism (VTE), the rate unusually high between 35 and 63% [1, 21, 29, 30]. Thrombosis is more venous than arterial, typically occurring early in the disease course with high recurrence risk and more common in patients who have cardiac and pulmonary inflammatory manifestations [21, 30, 31].

## Therapeutic considerations

Treatment of patients with VEXAS frequently requires a multi-pronged approach. Patients with VEXAS syndrome may have either a predominant hematologic or autoinflammatory presentation, which will drive specific therapy. At present most of the treatment regimens are based on single institution studies, observational studies, or case reports. Treatment is challenging since VEXAS is a markedly heterogeneous disease as evidenced in a cohort of patients in whom pathogenic *UBA1* mutations were identified with consistent disease penetrance, but much lower rates of severe inflammatory symptoms than previously reported [32]. Furthermore, the disease has increased risk of mortality and morbidity. Risk stratification of patients at diagnosis is critical in management. In patients with mild disease and no risk factors, we recommend treatment guided towards optimizing medical management and allo-HCT should be reserved for high-risk patients with refractory inflammatory disease or progressive bone marrow failure.

### Medical management/Non transplant options

*A. Control of inflammation*: There are several strategies to control inflammation in VEXAS. Most patients achieve clinical response with glucocorticoids, however the doses required for disease control are high and many will develop significant long-term steroid toxicities [1, 27, 29]. A number of steroid sparing agents have been used including anti-interleukin-1 [IL-1] inhibitors such as anakinra and canakinumab [1, 29, 33], anti-IL-6 inhibitors such as tocilizumab and siltuximab [29, 34, 35], JAK inhibitors such as ruxolitinib, tofacitinib, baricitinib, and upadacitinib [33, 36–38]and hypomethylating agents (HMA) in patients with MDS or MDS related cytogenetic mutations [10, 21, 39–41]. However, many patients remain unable to taper corticosteroids despite the use of these agents.

In a multi-institutional retrospective study, after 6 months of treatment with ruxolitinib, the complete clinical response was achieved in 87% of cases facilitating corticosteroid reduction or withdrawal compared to 11% for other JAK inhibitors (*p* = 0.002) [36]. However, many unanswered questions remain such as the timing of JAK inhibitors in the clinical course, duration of treatment, long-term and short-term toxicities including severe and opportunistic infections, hematologic toxicity and the need for specific prophylaxis and supportive care with these agents. While clinical efficacy was observed, two patients had progression of MDS and *UBA1* VAF increased with time despite ruxolitinib use, highlighting lack of disease modifying activity [36].

Other agents used in VEXAS include disease modifying antirheumatic drugs [DMARDs] such as low dose methotrexate, azathioprine, hydroxychloroquine, and mycophenolate mofetil (MMF) with very limited data on their efficacy and none have exhibited steroid sparing effect [10, 29, 42, 43]. TNFα inhibitors such as infliximab, adalimumab, golimumab, etanercept, certolizumab and IL-17 inhibitors such as secuzinumab and Ixekizumab have been tried with variable responses [10, 29, 33, 44]. The IL-6 inhibitor tocilizumab has a partial suppressive effect on the inflammatory response and in a systematic review, two thirds of the patients on tocilizumab were able to achieve glucocorticoid reduction but only 20% achieved complete response [43].

Supportive care is an important consideration in VEXAS. Those with significant cytopenias or MDS may require transfusions for anemia and thrombocytopenia. Additionally, VEXAS patients have lymphopenia and monocytopenia and majority of them are on glucocorticoid therapy, which will increase the risk for infections [45, 46]. Serious and life-threatening opportunistic infections including mycobacterial infections, recurrent skin infections, and pneumocystis jirovecii pneumonias (PJP) have been reported [47, 48]. Given the higher rates of opportunistic infections and long-term steroid use, patients are routinely prescribed antiviral and PJP prophylaxis [33]. Since VTE risk is high, patients who have had a VTE should remain on indefinite anticoagulation and prophylaxis should be considered in high-risk situations. This should be balanced against risk of bleeding, when thrombocytopenic and when on steroids.

### Management of MDS

#### Allogeneic Hematopoietic stem cell transplantation [Allo-HCT]

Allo-HCT is potentially curative treatment for VEXAS patients with hematologic disease or cytopenias [49]. Allo-HCT is designed to eradicate the *UBA1* clone which drives the clonal hematopoiesis in VEXAS. However, the affected individuals are older men, and the transplant related complications such as graft versus host disease (GVHD), organ toxicity, graft failure, and infectious complications are relatively high; thus, the risks must be weighted against the expected benefits. Careful pre-transplant screening is needed to establish the disease comorbidity to mitigate the transplant related morbidity and mortality (TRM).

##### Indications, patient selection, and timing

The major indication for allo-HCT in VEXAS is ineffective hematopoiesis and accompanying cytopenias. Selected patients with refractory inflammatory symptoms have also been successfully transplanted. In the United Kingdom (UK) series of the four patients who underwent an allo-HCT, three had severe and poorly controlled inflammatory illness; one patient had MDS [28]. In the updated Mayo clinic series recently presented at the Transplantation and Cellular Therapy (TCT) meeting in San Antonio, among the 10 patients who underwent an allo-HCT, 6 patients had steroid refractory inflammation, and the remaining 4 patients had either bone marrow failure or emerging myeloid neoplasm [50, 51]. The European Blood and Barrow Transplantation (EBMT) published their cohort of 19 patients who underwent allo-HCT, of these 13 patients had MDS, 5 had autoinflammatory manifestations and 1 had myeloproliferative disorder [52]. Since the majority of patients with VEXAS are older, with median age at diagnosis over 60 years, several questions remain regarding which patients benefit from an allo-HCT without increasing the risk of treatment related morbidity and TRM. Additional questions include what is the best timing to proceed to transplant, as many of the patients may present with potential end organ dysfunction due to prolonged immunesuppression. Current tools to assess transplant outcomes such as hematopoietic cell transplant - comorbidity index (HCT-CI) would place many patients at high risk due to multiple organ involvement and infections. Whether the HCT-CI adequately captures the risk and should this be used as a prognostic tool needs clarity from larger clinical trials [53]. To date there is no VEXAS specific disease activity score, nor prognostic models. We expect that as more transplant outcomes become available, there will be a more comprehensive model and risk stratification to assess these patients.

Patients with VEXAS syndrome display disease progression over time with declining performance status due to disease flares and from steroid induced complications. Therefore, we recommend allo-HCT early in the disease course before serious end organ injury makes them ineligible for transplant or investigational protocols. There are reports of VEXAS patients with associated hematological disease having higher mortality in the non-transplant setting, and this supports our recommendation for an early transplant in this subset of patients [27]. A crucial point is whether VEXAS patients need bridging therapy before allo-HCT and if so what type of therapy should they receive? what should be the status of MDS at the time of transplant? Does complete molecular remission prior to allo-HCT confer better prognosis?. Studies have shown VEXAS MDS is a low risk disease, and we believe achieving cytogenetic remission is less likely relevant than in standard MDS. All of these questions need to be answered in larger prospective studies, which should define transplant indications, timing, patient selection, and the role of bridging therapy. Currently, we believe in a patient centric approach based on the individual patient’s disease features, clinical condition, and organ dysfunction in decision making. The current and limited evidence suggests that using biologics, JAK inhibitors and HMA may serve as potential bridges to transplant enabling the patient to come to transplant with minimal hematological and inflammatory disease. There are case reports describing complete clearance of *UBAI* clone in patients outside the context of allo-HCT with HMA therapy [54]. In the French nationwide registry of 116 patients with VEXAS, of the 11 patients with concomitant MDS, clinical response was achieved in five patients (46%), suggesting azacitidine can be effective in selected patients with VEXAS and associated MDS [39]. However for severe cases of VEXAS syndrome with widespread multi-system involvement, including severe hematological abnormalities, allo-HCT holds promise for durable response and potential cure. Given the lack of established treatment guidelines, for now, we recommend early initiation of disease modifying agents or HMA therapy if associated MDS as a bridge before proceeding to allo-HCT.

##### Conditioning regimens and donor selection: myeloablative vs. reduced intensity

The goal of allo-HCT in VEXAS syndrome remains eradication of the *UBA1* clone and the associated inflammatory and hematological disease. To date only a small number of VEXAS allo-HCT cases and series have been reported (Table 1).

**Table 1:** Clinical trials and case reports of VEXAS patients who have undergone allo-HCT

Clinical study/case report	No. of patients (*n*)	Conditioning regimen(s)	Donor(s)	Stem cell source	GVHD prophylaxis	GVHD: acute and chronic	Clinical response	Other complications
French cohort [55]	6	Flu/Bu/ATG (2), Flu/Bu (2), Flu/Bu/Thiotepa (1), Bu/CP/ATG (1) [RIC]	MUD (4), MRD (2)	PBSC (5), BM (1)	CSA,/MMF (1), CSA,/MTX (3), CSA,/MMF/, CP (2)	Acute GVHD skin (1), skin and GI (2), cGVHD (2)	CR (5), Death (1) UBA1 clone status: NA	Bacteremia (catheter related; 4), BK virus hemorrhagic cystitis (1), CMV replication (1), Fusarium infection (1)
Mayo clinic cohort [50, 51]	10	Flu/Mel (7), Flu/Bu (2), Bu/Flu/Thiotepa (1)	MUD (5), MRD (4), Haplo (1)	PBSC (9), BM (1)	PTCy/Tac/MMF (9), Tac/MTX (1)	Late skin GVHD (2)	No deaths at 9 month f/u. Day 100 chimerism mixed (> 10% recipient, myeloid *n* = 4, lymphoid *n* = 5), no relapse. At 12 months ( *n* = 5): No disease, BM morphologic CR and UBA1 mutation clearance in 4.	Mucositis (2/5)[50], infections (*n* = 6)[51], lactic acidosis (1/5)[50], C.diff diarrhea (1/5)[50], skin rash drug induced (2/5)[50]
UK Cohort [28]	4	Flu/Bu/Thiotepa (1), Flu/Mel/Alemtuzumab (1), Flu/Treo/Alemtuzumab (1), Flu/Bu/ATG (1)	Haplo (1), MUD (2), MSD (1)	PBSC (4)	CSA/Tac/MMF (1), CSA (1), CSA/MMF/Alemtuzumab (1), ATG/CSA (1)	Grade 1 GVHD (acute) (1)	Alive-2/4 Chimerism: CD3 99% and 100% and CD33 100%. UBA1 clone: NA	Salmonella and pseudomonas sepsis, multiorgan failure (1), HLH, aseptic encephalitis (1), metabolic acidosis, myelitis, optic neuropathy (1).
Case report [59]	1	Flu/thiotepa/melphalan/ATG [MA]	MMUD (9/10)	T cell depleted PBSC (TCR αβ + and CD19+	MMF and prednisone	None	Alive, CR chimerism: 100% donor UBA1 clone: NA	Mucositis, CMV colitis,
Case Report [57]	1	Flu/Bu [RIC]	MMUD	PBSC	PT Cy (days + 3/+4), CSA and MMF	Acute: skin, stage 1, acute GI GVHD	Alive, CR, and UBA1 mutation not detected	Left wrist swelling, cause unknown
EBMT multicenter study [60]	19	RIC-74%, MAC − 26% Fludarabine based; Flu/Bu (*n* = 7), treosulfan (*n* = 4), Bu/Thiotepa (*n* = 3), melphalan ( *n* = 2), 1 received TBI), melphalan and thiotepa (*n* = 1), FLAMSA/Bu- Cy sequential combination (*n* = 2).	MSD: 16%, MUD: 63% MMRD: 16% MMUD: 5%	PBSC:18 BM:1	PT Cy [day + 3/+4] *n* = 6, ATG [*n* = 11] or alemtuzumab [*n* = 2 ] + Cyclo/methotrexate [*n* = 7] or MMF [*n* = 5]. Exvivo TCRαβ/CD19 depleted graft (*n* = 1).	Grade II-IV acute GVHD: 26% Chronic extensive GVHD: 4	2 -year OS: 74.2% at 14 months from allo-HCT, TRM: 25.8% 94% full donor chimerism.*UBA1* disappearance in 6 patients.	4 deaths, 3 died due to infection and 1 from CNS toxicity.

NA: Not available/Not applicable; Bu: Busulfan; Flu: Fludarabine; MMF: Mycophenolate Mofetil, TBI: Total Body Irradiation; CP: Cyclophosphamide; PT Cy: Post Transplant Cyclophosphamide; MUD: Matched Unrelated Donor, MRD: Matched Related Donor; MMUD: Mismatched Unrelated Donor, MMRD: Mismatched Related Donor, MAC: Myeloablative Conditioning, RIC: Reduced Intensity Conditioning; ATG: Antithymocyte Globulin; CSA: Cyclosporine; Tac: Tacrolimus; Allo-HCT: Allogeneic Hematopoietic Cell Transplantation; CMV: Cytomegalovirus; HLH: Hemophagocytic Lymphohistiocytosis; C.diff: Clostridium difficile; Haplo: Haploidentical Donor; MDS: Myelodysplastic Syndrome, TRM: Treatment Related Mortality; GVHD: Graft versus Host Disease; GI: Gastrointestinal; CR: Complete Remission

In a UK series of 4 VEXAS patients, the preparative regimens included Fludarabine (Flu)/Busulfan(Bu)/thiotepa, Flu/Melphalan(Mel)/alemtuzumab, Flu/Treosulfan/alemtuzumab and Flu/Bu/Antithymocyte globulin (ATG). Two patients received stem cells from matched unrelated donors, one from a matched sibling and the other underwent haploidentical donor transplant [28]. The transplant outcomes were variable, with one of the four alive at five months and another alive and in remission at 40 months post-transplant. Of the two deaths, one died from sepsis and multiorgan failure 11 days post-transplant and the second died from infectious complications at 11 months from transplant [28]. Two French VEXAS groups describe a total of 7 patients with MDS in 6 and one with myelofibrosis. The conditioning regimens were reduced intensity with Bu/Flu (*n* = 3), Bu/Flu/ATG ( *n* = 2), Bu/Cyclophosphamide (Cy)/thiotepa (*n* = 1) and Bu/Cy/ATG ( *n* = 1). Six patients are alive at the time of reporting, three of them > 30 months, and the other three < 6 months while 1 patient died from infection. One of the seven patients cleared the *UBA1* mutation, and all patients were off therapy for VEXAS [55–58]. In the Mayo clinic series, all ten patients received reduced intensity conditioning (RIC) with Flu/Mel ( *n* = 7), Bu/Flu (*n* = 3) and Bu/Flu/Thiotepa (*n* = 1) who underwent haploidentical transplant. Four patients received T cell replete peripheral blood stem cells (PBSCs)from matched related donors and 5 received PBSCs from matched unrelated donors [50, 51]. At a median follow up of 9 months from transplant there were no deaths reported, and among patients with > 12 months of follow up (*n* = 5), there was no evidence of disease, normalization of bone marrow morphology and inflammatory markers and *UBA1* mutation clearance in peripheral blood in 4 patients. They observed that mixed chimerism( either in lymphoid or myeloid fraction) was not associated with disease relapse or graft failure and resolved after withdrawal of immunesuppression [50, 51]. In the EBMT series of 19 patients, RIC regimen was used in 14 patients and myeloablative conditioning (MA) in 5 patients. Overall conditioning was based on a backbone of Flu / Bu ( *n* = 7), treosulfan (*n* = 4), Bu and thiotepa (*n* = 3), Mel (*n* = 2, of whom 1 patient received total body radiation), Mel and thiotepa (*n* = 1) and FLAMSA/Bu- Cy sequential combination in 2 patients [52]. T cell replete PBSCs were the stem cell source in 18 patients and 1 patient received an ex vivo manipulated graft after TCR αβ/CD19 depletion. Donors consisted of matched unrelated donors (63%), matched siblings (16%), mismatched related donors (16%) and mismatched unrelated donors ( 5%). Overall, 94% of patients achieved full donor chimerism at last follow up and in 2 patients donor lymphocyte infusion were administered because of an initial (< 6 months) mixed chimeric state. All, but one patient experiencing primary graft failure, reached neutrophils and platelet engraftment at a median of 16 days ( range, 8–32) and 15 days ( range, 4–47) from allo-HCT. At a median follow up of 14 months from allo-HCT, 2-year overall survival (OS) was 74.2% and TRM was 25.8%. Of the 4 deaths, 3 patients died of bacterial infection and 1 from CNS toxicity [52].

Conditioning regimens represent an important variable in all allo-HCT outcomes. As detailed above in the listed case series, the majority of the patients received RIC regimens. However, the ideal conditioning regimen with least non relapse mortality (NRM), TRM and eradication of clone is yet to be determined. Currently there are two ongoing studies investigating the role of allo-HCT in VEXAS patients, one in the USA and one in Europe [Table 2]. The USA trial is led by the National Institute of Health (NIH) and is a phase 2 prospective trial [ NCT 05027945] consisting of 2 cohorts of 17 patients in each cohort. The conditioning regimen will be reduced intensity with Bu for three days and Flu for four days in cohort A (matched donors). The cohort B will include mismatched and haploidentical related donors; conditioning regimen will be with Bu for two days, Flu for four days, low dose Cy for two days and single dose total body radiation (TBI) of 200 cGy. Post-transplant Cy on days + 3 and + 4 post stem cell infusion followed by sirolimus with mycophenolate mofetil (MMF) starting on day + 5 will be used for GVHD prophylaxis in both arms. The European trial will be run by the chronic malignances and autoimmune disease working group of EBMT with collaboration of the rheumatology advisory board. They plan a retrospective analysis of VEXAS MDS patients who underwent an allo-HCT across all centers in Europe. They then plan to conduct a prospective observational study to evaluate the role of allo-HCT with a focus on MDS in VEXAS patients. The primary objectives would include OS and event free survival (EFS). Secondary objectives will include TRM, graft versus host disease (GVHD) and VEXAS specific symptoms.

**Table 2:** Ongoing Allo-HCT clinical trials

Clinical trial	Trial design	Conditioning/GVHD protocol	Patient population	Current status	Outcomes
USA; NCT05027945	Phase II, interventional, prospective	Arm A: Bu/Flu Post transplant CP, MMF, sirolimus [MUD/MRD] Arm B: Flu/Low dose CP/TBI 200 cGy/busulfan [MMUD/Haplo], Post transplant CP/MMF/Sirolimus	Adults 18–75	enrolling	Evaluate allo-HCT in patients with VEXAS and improve disease outcome. Sustained donor engraftment; reversal of the VEXAS clinical phenotype at 1 and 2 year post allo-HCT
Europe: EBMT	Retrospective	NA	Adult patients	NA	Identify total number of cases of VEXAS cases, MDS co-diagnosis and identify characteristics of transplanted patients across EBMT center
	Prospective, observational	NA	NA	NA	Role of allogeneic HSCT with a focus on VEXAS/MDS clinical dyad. Objectives include OS and EFS, incidence of TRM, GVHD and disease specific outcomes.

NA: Not available/Not applicable; Bu: Busulfan; Flu: Fludarabine; MMF: Mycophenolate Mofetil, TBI: Total Body Irradiation; MUD: Matched Unrelated Donor, MRD: Matched Related Donor; MMUD: Mismatched Unrelated Donor, Allo-HCT: Allogeneic Hematopoietic Cell Transplantation; Haplo: Haploidentical Donor; MDS: Myelodysplastic syndrome, TRM: Treatment Related Mortality; GVHD: Graft versus Host Disease; EBMT: European Society for Blood and Marrow Transplantation

##### Graft Versus host disease [GVHD] prophylaxis

The incidence of GVHD varies in allo-HCT studies. The ideal GVHD prophylaxis would decrease the incidence and severity of both acute and chronic GVHD. In the French study, patients received cyclosporine, MMF, cyclophosphamide and methotrexate. Four patients developed acute GVHD involving skin (grade 1), and GI (maximum grade III). Two patients developed chronic GVHD involving the skin and liver [55–57]. In the UK series, the GVHD prophylaxis included cyclophosphamide, tacrolimus, MMF, cyclosporine, alemtuzumab and ATG. Two of the four patients developed GVHD [28]. In the Mayo clinic series, 9 patients received post-transplant cyclophosphamide, tacrolimus and MMF and one patient received tacrolimus and methotrexate ( due to cardiac dysfunction). They observed late acute skin GVHD in 2 patients, and none developed GI or liver acute GVHD, and no patient has developed chronic GVHD at the time of reporting. All patients remained on steroids through transplant until day + 100 [50, 51]. In the EBMT cohort, GVHD prophylaxis included post-transplant cyclophosphamide (*n* = 6), or serotherapy (ATG *n* = 11, alemtuzumab *n* = 2) in combination with cyclosporine and short course methotrexate (*n* = 7) or MMF (*n* = 5). Acute GVHD occurred in 58% of cases with grade 2 to 4 noted in 26% of patients at a median of 1.9 months ( range, 0.3-4) from graft infusion. Chronic GVHD occurred in 4 patients at a median of 4.6 months ( range, 3.3-5) from transplant, which in 2 cases resolved by the first year from allo-HCT. One patient continues to have severe, multiple refractory chronic GVHD at last follow up and one patient died in CR due to bacterial infection [52]. The prospective NIH clinical trial will use post-transplant cyclophosphamide, sirolimus and MMF as GVHD prophylaxis for both the patient cohorts. As of now the GVHD and relapse free survival (GRFS) favors using post-transplant cyclophosphamide and tacrolimus and MMF but needs confirmation in larger studies.

##### Monitoring/chimerism analysis

Optimal monitoring of patients in the post-transplant period for both groups of patients, those who were transplanted for MDS or for refractory inflammatory symptoms is ill defined. Bone marrow evaluations need to be performed as per the MDS/AML guidelines on day 100 post-transplant, and at 12 months. Whether earlier BM evaluations are needed and if so, how often, the role and timing of chimerism in determining long term success after transplant and whether mixed chimerism is sufficient for cure [51], particularly in the inflammatory phenotype, all remain unclear and need to be addressed in prospective trials. Defining remission in patients transplanted for inflammatory phenotype is difficult, as it is unclear whether to consider patients off steroids or other immunosuppressive therapy to be in remission or whether reversal of end organ damage from VEXAS syndrome is to be considered as remission.

##### Long term follow up

Long term follow- up of patients who undergo allo-HCT is a challenge. These patients not only have complications from allo-HCT, but they have long term toxicities from primary treatment for VEXAS syndrome particularly the steroid toxicity. Patients require a multidisciplinary team approach including the transplant physician, rheumatologist, primary care physicians along with occupational and physical therapists, and other ancillary staff. The team should include all other specialists who are involved in personal care of these patients such as endocrinologist to manage steroid induced diabetes and osteoporosis, orthopedic or the spine surgeons, gastroenterologist, dermatologist, ophthalmologist, oral surgeons and psychiatrist and behavioral therapists. Ultimately the team should address not just the transplant associated long term toxicities but also the primary disease related and treatment related toxicities.

## Conclusion

VEXAS syndrome is a hemato-inflammatory syndrome with heterogeneous and challenging presentations ranging from predominantly inflammatory manifestations to severe involvement of bone marrow with myelodysplasia. Patients are complex and require multidisciplinary evaluation and management. There are no current standard guidelines in the management of these patients, and they have a high morbidity and mortality from the disease and from treatment. Although allo-HCT appears to be a promising curative option, the unknown variables include a lack of standardized transplant indications, optimal conditioning regimens, best post-transplant care and timing of transplant. There is a significant unmet need for prospective trials in VEXAS to better define the role of transplant and to assess its utility and long-term outcomes.

## Data Availability

No datasets were generated or analysed during the current study.
